# *Gnidia glauca* flower extract mediated synthesis of gold nanoparticles and evaluation of its chemocatalytic potential

**DOI:** 10.1186/1477-3155-10-17

**Published:** 2012-05-01

**Authors:** Sougata Ghosh, Sumersing Patil, Mehul Ahire, Rohini Kitture, Deepanjali D Gurav, Amit M Jabgunde, Sangeeta Kale, Karishma Pardesi, Vaishali Shinde, Jayesh Bellare, Dilip D Dhavale, Balu A Chopade

**Affiliations:** 1 Institute of Bioinformatics and Biotechnology, University of Pune, Pune, 411007, India; 2 Department of Electronic Science, Fergusson College, Pune, 411004, India; 3 Department of Chemistry, Garware Research Centre, University of Pune, Pune, 411007, India; 4 Department of Applied Physics, Defense Institute of Advanced Technology, Girinagar, Pune, 411025, India; 5 Department of Microbiology, University of Pune, Pune, 411007, India; 6 Department of Chemical Engineering, Indian Institute of Technology, Bombay, Powai, Mumbai, 400076, India

**Keywords:** *Gnidia glauca*, Gold nanoparticles, UV-Visible spectroscopy, Transmission electron microscopy, High resolution transmission electron microscopy, Elemental mapping, Energy dispersive spectroscopy, Dynamic light scattering, X-ray diffraction, Chemocatalysis

## Abstract

**Background:**

Novel approaches for synthesis of gold nanoparticles (AuNPs) are of utmost importance owing to its immense applications in diverse fields including catalysis, optics, medical diagnostics and therapeutics. We report on synthesis of AuNPs using *Gnidia glauca* flower extract (GGFE), its detailed characterization and evaluation of its chemocatalytic potential.

**Results:**

Synthesis of AuNPs using GGFE was monitored by UV-Vis spectroscopy and was found to be rapid that completed within 20 min. The concentration of chloroauric acid and temperature was optimized to be 0.7 mM and 50°C respectively. Bioreduced nanoparticles varied in morphology from nanotriangles to nanohexagons majority being spherical. AuNPs were characterized employing transmission electron microscopy, high resolution transmission electron microscopy. Confirmation of elemental gold was carried out by elemental mapping in scanning transmission electron microscopic mode, energy dispersive spectroscopy and X-ray diffraction studies. Spherical particles of size ~10 nm were found in majority. However, particles of larger dimensions were in range between 50-150 nm. The bioreduced AuNPs exhibited remarkable catalytic properties in a reduction reaction of 4-nitrophenol to 4-aminophenol by NaBH_4_ in aqueous phase.

**Conclusion:**

The elaborate experimental evidences support that GGFE can provide an environmentally benign rapid route for synthesis of AuNPs that can be applied for various purposes. Biogenic AuNPs synthesized using GGFE exhibited excellent chemocatalytic potential.

## Background

Nanomaterials of various shapes and sizes have been the subject of utmost interest due to their potential applications in industries, biomedical diagnostics and electronics, over the past decade [1-10]. Most of the available chemical processes for synthesis of gold nanoparticles (AuNPs) involve toxic chemicals that get adsorbed on the surface, leading to adverse effects in medical applications. Presently there is a growing need to develop environmentally benign process for rapid synthesis of nanoparticles [11].

At present, biological methods have an increasing interest because of the necessity to develop new clean, cost-effective and efficient synthesis techniques. Lately, many biological systems such as bacteria, yeast, fungi and several plant extracts have been investigated due to their ability to reduce metal ions and form nanoparticles [12-22]. Synthesis of nanoparticles employing plants can potentially render more biocompatibility to the nanoparticles [23-25].

**Figure 1:**
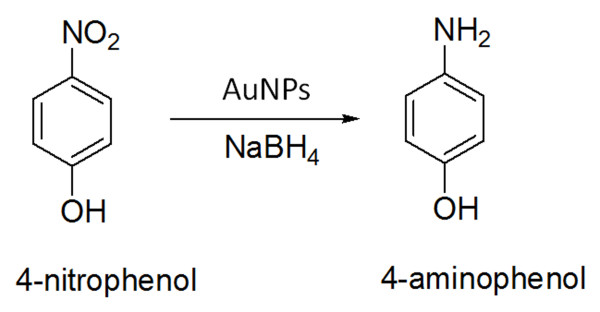
Scheme for chemical catalysis from 4-nitrophenol to 4-aminophenol.

Plant mediated synthesis of metal nanoparticles is gaining more importance owing to its simplicity, rapid rate of synthesis of nanoparticles of attractive and diverse morphologies and elimination of elaborate maintenance of cell cultures and eco-friendliness. It is reported that *Achillea wilhemsii* flowers can be used for synthesis of highly stable AuNPs [26]. AuNPs has drawn special attention owing to its immense importance in biomedical and chemical applications [27,28]. Among the array of applications, the reduction of 4-nitrophenol (4-NP) to 4-aminophenol (4-AP) using noble metal nanoparticles as catalysts has become one of the significant model reactions (Figure 1). AuNPs can effectively catalyze the reduction of nitro compounds by the electron transfer from donor BH_4_^–^ to acceptor nitro groups. The reduction process is monitored through measuring the change of absorbance at 400 nm as a function of time.

In this work, we have investigated the biosynthesis of AuNPs using *Gnidia glauca* flower extract (GGFE) as a clean technology. *G. glauca* is an endemic flora of Western Ghats of India. It has an array of medicinal applications in sore throat, abdominal pain, wounds, burns, and snake bites, contusions, swellings, back ache, and joint ache [29,30]. Recently, we have reported its antidiabetic property [31]. However, there are no reports till date that documents its potential in nanobiotechnology to synthesize nanoparticles and thereby evaluating its chemocatalytic applications.

Herein, we report the biogenic synthesis of AuNPs using aqueous extract of *G. glauca* flower for reduction of Au^3+^ ions. We also investigated the effects of reaction conditions such as time course, reaction temperature and concentration of chloroauric acid on the rate of synthesis of the AuNPs. Further, we demonstrated its chemocatalytic potential in reduction of 4-nitrophenol (4-NP) to 4-aminophenol (4-AP).

## Results and discussion

### Biosynthesis of AuNPs by GGFE

**Figure 2:**
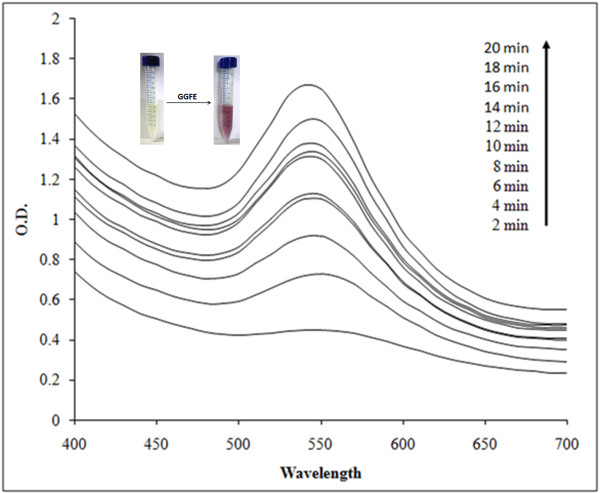
UV-vis spectra recorded as a function of reaction time of 1 mM chloroauric acid solution with GGFE at 40°C.

**Figure 3:**
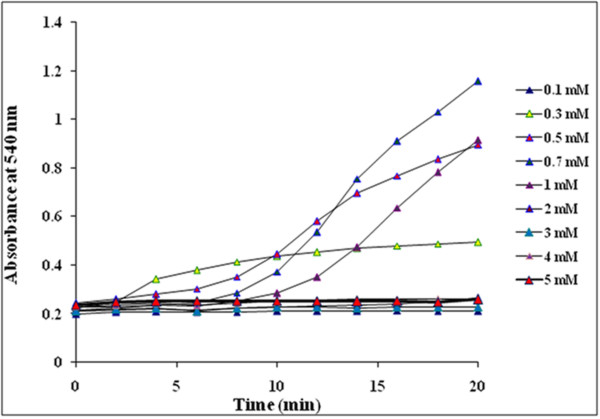
Time course of AuNPs formation obtained with different concentrations of chloroauric acid using GGFE at 40°C.

**Figure 4:**
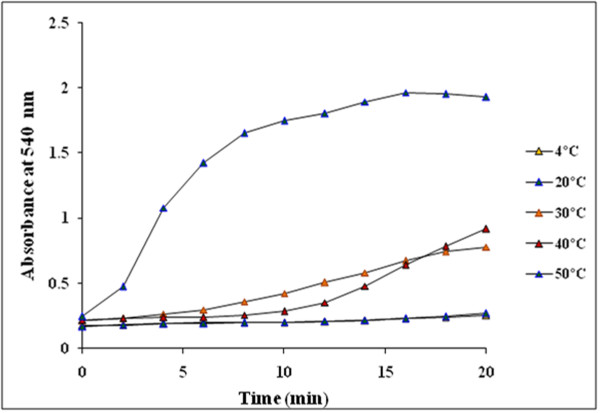
Time course of AuNPs formation obtained with 1 mM chloroauric acid using GGFE at different reaction temperature.

Reduction of Au^3+^ to AuNPs by GGFE could be followed by color change from yellow to ruby red (Figure 2) and further by UV-vis spectroscopy. The peak observed at 540 nm confirmed the synthesis of AuNPs as it is in agreement with the previous reports [11]. Though initially there was no significant peak at 2 min but at 4 min the building of peak at 540 nm marked the initiation of synthesis of AuNPs. Subsequent rise in peak with a maximum at 20 min supported that the reported route of AuNPs synthesis is novel as well as rapid as compared to *Mirabilis jalapa* flower wherein the synthesis was reported to be completed in 2 h [32]. Similarly, it was found to be faster even as compared to black tea extract [33]. Optimization studies showed that 0.7 mM of chloroauric acid facilitated maximum synthesis of AuNPs as compared to other concentrations (Figure 3). The effect of concentration of chloroauric acid on the kinetics of the synthesis of AuNPs was found to be prominent as although the rate of synthesis increased with concentration till 1 mM, at higher concentration there was no synthesis. Temperature optimization revealed a direct effect on the reaction kinetics (Figure 4). Synthesis failed to be initiated at lower temperatures like 4 and 20°C while a moderate rate of reaction was observed at 30 and 40°C. The rate of reaction was found to be maximum at 50°C which supported the fact that higher temperature plays a key role in enhancing the reaction rate which is in well agreement with synthesis of AuNPs mediated by *Nyctanthes arbortristis* flower extract [34].

### TEM, HRTEM and DLS

**Figure 5:**
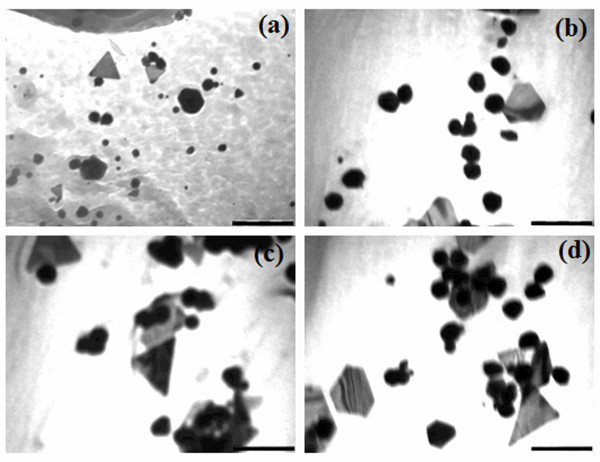
**Characterization of AuNPs formed with 1 mM chloroauric acid and 5 % GGFE at 40°C by transmission electron microscopy (TEM).** (**a**) Anisotropic gold nanotriangles; (**b**) Gold nanotrapezoids; (**c**) Gold nanospheres and triangles with equilateral edges ; (**d**) Gold nanotrapezoids and spherical nanoparticles adsorbed on surface of triangles. The inset bars represent 200 nm.

**Figure 6:**
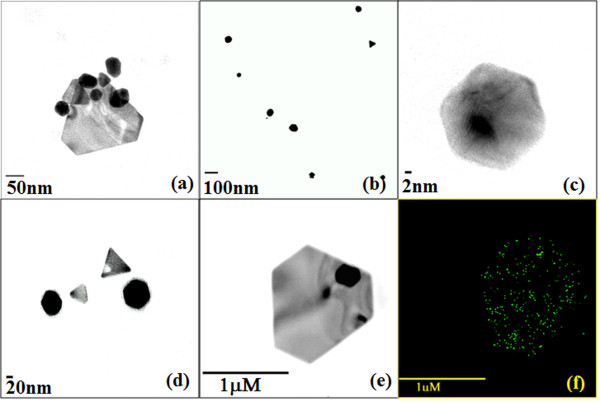
**Characterization of AuNPs formed with 1 mM chloroauric acid and 5 % GGFE at 40°C by high resolution transmission electron microscopy (HRTEM).** (**a**) Anisotropic AuNPs adsorbed on surface of each other; (**b**) Spherical nanoparticles in a range of 10 nm.; (**c**) Gold nanohexagon with equilateral edges; (**d**) Gold nanotriangles and hexagons ; (**e**) Scanning transmission mode micrographs for gold nanotrapezoid.; (**f**) Elemental mapping of gold nanotrapezoid.

The size and shape of the bioreduced AuNPs were elucidated with the help of TEM. TEM images in (Figure 5) confirm the formation of AuNPs. The figure nicely illustrates that most of the particles were spherical in a range of 5 to 20 nm as was reported in case of other plant extracts as well [35,36]. Pronounced anisotropy among the bioreduced nanoparticles was evident from the nanotriangles and nanoprisms (Figure 5a) which is similar to *Rosa hybrida* petal extract which is reported to synthesize polydispersed AuNPs [37]. Shape evolution, particularly triangles are attributed owing to the chloride ions contributed by the chloroauric acid [11]. In some cases nanoparticles were found in small aggregates adsorbed onto the surface of the particles with larger dimensions between 50 to 150 nm (Figure 5d). Similarly, HRTEM images provided valuable information about the architecture of the biogenic AuNPs. Figure 6a shows the anisotropy among the bioreduced nanoparticles and their positioning by attachment on the surface of each other. Very small nanospheres could be spotted in Figure 6b. Spherical nanoparticles are reported to be formed at low extract concentration when the extract has excellent capping and stabilizing property [36]. Figure 6c and d show the fine nanostructure of the hexagons and triangles with equilateral edges. Elemental mapping on scanning transmission electron microscopic mode confirmed the bioreduced nanoparticles employing GGFE was elemental gold (Figure 6f).

**Figure 7:**
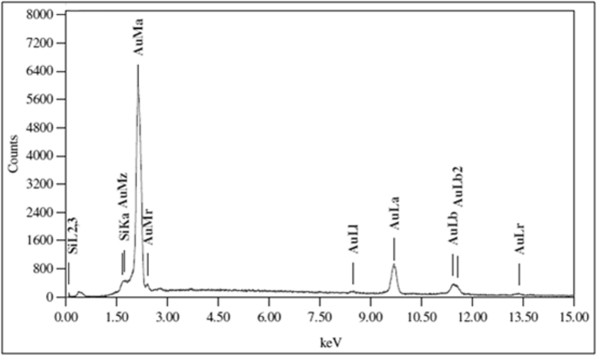
Representative spot EDS profile confirming the presence of AuNPs.

**Figure 8:**
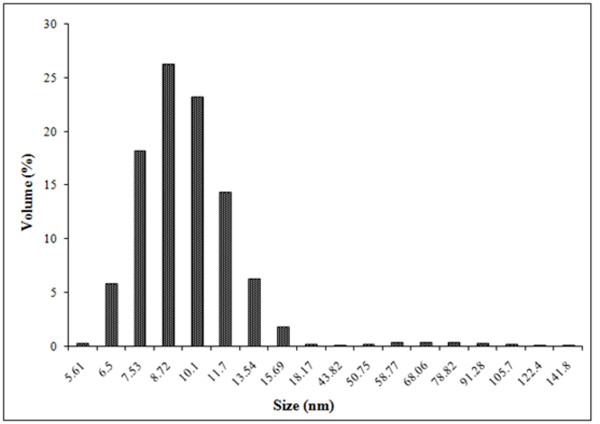
A histogram of size distribution of AuNPs synthesized by GGFE.

In the analysis of synthesized particles by energy dispersive spectroscopy (EDS), the presence of elemental gold signal was confirmed (Figure 7). Particle size distribution of the AuNPs determined by dynamic light scattering is shown in Figure 8 which was found well in agreement with TEM and HRTEM analysis.

### XRD analysis

**Figure 9:**
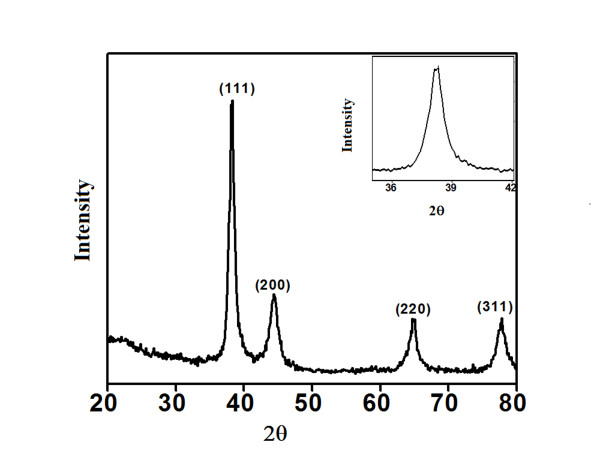
Representative XRD profile of thin film AuNPs.

The phase formation of the synthesized AuNPs was analysed employing X-ray diffraction which confirmed that the bioreduced metal nanoparticles are of elemental gold (Figure 9). Existance of peaks (111), (200), (220) and (311) matched with the standard Joint Committee for Powder Diffraction Set (JCPDS) data- 04784. This confirmed face centered cubic structured AuNPs formation. Peak broadening indicated restricted particle size. Enlarged pattern of (111) peak is shown in the inset of XRD plot. The crystallite size was calculated using Scherrer’s formula

(1) $$d=\frac{0.9\lambda }{\beta \cos\theta }$$

Here 0.9 is the shape factor, generally taken for a cubic system, λ is the x-ray wavelength, typically 1.54 Å, β is the full width at half the maximum intensity (FWHM) in radians, and θ is the Bragg angle. Using the above formula the crystallite size calculated is ~10 nm.

### Fourier Transform Infrared Spectroscopy (FTIR) analysis

**Figure 10:**
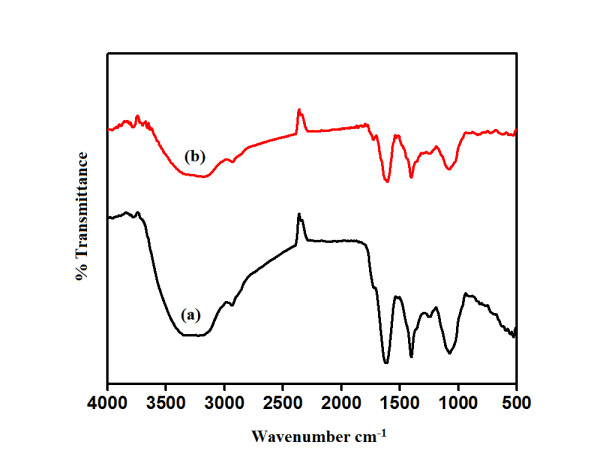
FTIR absorption spectra of dried GGFE before bioreduction (a), after complete bioreduction at 50°C (b) of chloroaurate ions.

FTIR absorption spectra GGFE before and after reduction of Au^3+^ are shown in Figure 10. GGFE before bioreduction (Figure 10a) shows strong peak at ~3300 cm^-1^ which is a characteristic of hydroxyl group in alcoholic and phenolic compounds. Free water soluble flavonoids are reported to play a major role in the biogenic synthesis and stabilization of AuNPs employing *Syzygium aromaticum* flower [38,39]. The sharp peak at 1607 cm^-1^ along with other at ~1450 cm^-1^ represents C=C characteristic group of phenols [40]. Bonds in the range 1220-1240 cm^-1^ represent aliphatic amines. This gives evidence of existence of amine group in GGFE. Existence of alcohol group was supported by the peak at ~1100 cm^-1^. These four peaks present before and after synthesis of nanoparticle may also indicate that GGFE may play a role in stabilizing AuNPs by adsorbing on the surface of bioreduced AuNPs. A small peak shift at ~2930 cm^-1^ may indicate bonding of extract to the AuNPs. C–H stretches of alkanes appear from 3000-2850 cm^-1^ may be due to bonding of alkanes present in GGFE to AuNPs.

### Chemoctalytic property of AuNPs

**Figure 11:**
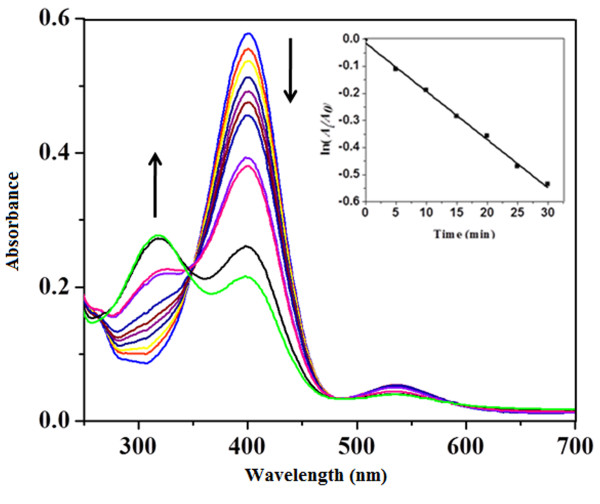
**Time dependent UV–vis spectra for monitoring*4*-nitrophenol reduction by NaBH**_**4**_**catalyzed by AuNPs.** The inset shows the plot indicating the variation of ln (A_t_/A_0_) vs time.

In alkaline NaBH_4_ medium, the absorption peak at 317 nm for 4-NP showed bathochromic shift at 400 nm due to formation of 4-nitrophenolate ion. As the reduction reaction does not proceed without catalyst, the peak (400 nm) due to 4- nitrophenolate remained unaltered. After addition of catalytic amount of AuNPs, the peak at 400 nm due to 4-NP decreased in intensity while a new peak at 300 nm appeared due to formation of 4-AP. This observation showed that the AuNPs catalyzed the reduction reaction which is primarily a size and shape dependent phenomena [41-43]. As the concentration of NaBH_4_ used was much higher than that of 4-NP, the order of the reaction was considered to be pseudo-first order reaction which is in agreement with Gangula *et al*[44]. Inset of Figure 11 shows a good linear correlation of ln (*A*_*t*_*/A*_*0*_) versus time and the kinetic rate reaction constant was estimated to be 1.78 × 10^-2^ min^-1^. (*A*_*t*_: absorbance at 400 nm at time *tA*_0_: absorbance at 400 nm at *t* = 0).

## Conclusion

In conclusion GGFE mediated synthesis of AuNPs has been demonstrated to be a rapid and environmentally benign route. Variation of reaction conditions had pronounced effect on the reaction kinetics. Optimum conditions for maximum synthesis were found to be 0.7 mM of chloroauric acid at 50°C. AuNPs with exotic shapes like nanoprisms, nanotriangles, hexagons trapezoids were synthesized. Spherical nanoparticles were in abundance which were found to be face centered cubic (FCC) structured gold (111). Bioreduced AuNPs showed excellent catalytic properties in a reduction reaction of 4-nitrophenol to 4-aminophenol by NaBH_4_ in aqueous phase. Thus this rapid, eco-friendly and economical route can be used to synthesize AuNPs with wide biotechnological and chemical applications.

## Materials and methods

### Plant material and preparation of extract

*G. glauca* flowers were collected from Western Ghats of Maharashtra, India. The flowers were thoroughly washed in running tap water for 15 min and then shade dried for 2 days at room temperature. Dry flowers were ground into fine powder in an electric blender. 5 g of this powder was suspended in 100 mL of distilled water in a 300 mL Erlenmeyer flask followed by boiling for 5 min before finally decanting it. The extract obtained was filtered through Whatman filter paper No.1. The filtrate was collected and stored at 4°C which was used throughout all the experiments.

### Synthesis of gold nanoparticles

Reduction of Au^3+^ ions was initiated by addition of 5 mL of GGFE to 95 mL of 10^-3^ M aqueous chloroauric acid solution in a 500 mL Erlenmeyer flask. The pH of the extract was found to be neutral. Thereafter, the flasks were shaken at a rotation rate of 150 rpm in the dark at 40°C. Reduction of the Au^3+^ ions was monitored by measuring the UV-vis spectra of the solution at regular intervals on a UV-1650CP Schimadzu spectrophotometer operated at resolution of 1 nm. Effects of temperature and concentration of chloroauric acid on the rate of AuNPs were studied by carrying out the reaction in water bath at 4-50°C with reflux and by varying the concentration of chloroauric acid from 0.1-5 mM.

### Transmission Electron Microscopy (TEM), High Resolution Transmission Electron Microscopy (HRTEM) and Dynamic Light Scattering (DLS) measurements

Morphology of the bioreduced AuNPs was studied by transmission electron microscopy (Tecnai 12 cryo TEM, FEI, Netherland). Further the size and shape of the AuNPs were characterized by JEOL-JEM-2100 higher resolution transmission electron microscope (HRTEM) coupled with elemental composition mapping under scanning transmission electron microscopic mode (STEM). Energy dispersive spectra of AuNPs was taken in the energy dispersive spectrometer (EDS) equipped in JEOL JSM 6360A analytical scanning electron microscope at an energy range 0-20 keV confirmed the synthesis of AuNPs using GGFE. The size of particles was analyzed by using the dynamic light scattering equipment (Zetasizer Nano-2590, Malvern Instruments Ltd, Worcestershire, UK) in 3 mL of reaction mixture in a polysterene cuvette.

### X ray diffraction (XRD) measurements

The phase formation of bio-reduced AuNPs was studied with the help of XRD. The diffraction data of thoroughly dried thin films of nanoparticles on glass slides was recorded on D 8 Advanced Brucker X ray diffractometer with Cu Kα (1.54 Å) source.

### Fourier Transform Infrared (FTIR) spectroscopy

AuNPs synthesized after 20 min of reaction between 1 mM chloroauric acid solution and GGFE were centrifuged at 10,000 rpm for 15 min at room temperature, following which the pellet was redispersed in sterile distilled water to remove any uncoordinated biological molecules. In order to ensure better separation of free entities from the nanoparticles, the process of centrifugation and redispersion in sterile distilled water was repeated thrice. The purified pellet was then dried and subjected to FTIR (Shimadzu IR Affinity) spectroscopy measurement using the potassium bromide (KBr) pellet technique in the diffused reflection mode at a resolution of 4 cm^-1^. Au nanoparticle powder was mixed with KBr and subjected to IR source 500-4000 cm^-1^. Similar process was used for the FTIR study of GGFE before and after bioreduction.

### Catalytic reduction of 4-nitrophenol

The catalytic reduction of 4-NP was studied in a standard quartz cuvette by adding 0.8 mL of aqueous NaBH_4_ solution (1.0 mM) to 1.0 mL of 4-NP aqueous solution (0.1 mM). Then 200 μL of aqueous suspension of AuNPs (0.1 mM) was introduced into the solution and time dependent absorption spectra were recorded after every 5 min in the range of 200-800 nm at 25°C. The progress of reaction was monitored by UV- visible spectrophotometer as both the starting material, 4-NP and the product 4-AP shows a different absorption in the UV-visible region.

## Competing interests

The authors declare that they have no competing interests.

## Authors’ contributions

SG carried out the synthesis UV-Vis spectroscopic experimentation, optimization of temperature and concentration and data interpretation. SP designed the experimentation and data interpretation and manuscript writing of HRTEM and elemental map analysis. MA carried out the plant selection, processing, extract preparation and determination of size by DLS and data interpretation. RK and SK carried on the FTIR and XRD analysis, interpretation and manuscript writing. VSS and DDG conceived the idea and designed the study for the chemical catalysis and its detailed data interpretation. DDD, JAM, BAC and KP concieved the study, participated in its design, coordination, scientific drafting, editing and correcting the manuscript. All authors read and approved the final manuscript.
